# Comparing Ultrasound‐Guided Injection of Oxygen‐Ozone vs. Corticosteroid vs. Prolotherapy in the Treatment of Shoulder Tendinopathy: A Prospective, Multicenter Randomized Clinical Trial

**DOI:** 10.1155/prm/1582085

**Published:** 2026-09-29

**Authors:** Arash Babaei-Ghazani, Naseh Yousefi, Bijan Forogh, Lobaneh Janbazi, Tannaz Ahadi, Hosna Soleymanzadeh, Fatemeh Jahanjoo, Hamid Reza Fadavi, Farzane Rouzegari

**Affiliations:** ^1^ Department of Physical Medicine and Rehabilitation, Neuromusculoskeletal Research Center, School of Medicine, Iran University of Medical Sciences, Tehran, Iran, iums.ac.ir; ^2^ Department of Physical Medicine and Rehabilitation, University of Montreal Health Center, Montreal, Quebec, Canada; ^3^ Department of Physical Medicine and Rehabilitation, Physical Medicine and Rehabilitation Research Center, Tabriz University of Medical Sciences, Tabriz, East Azerbaijan, Iran, tbzmed.ac.ir; ^4^ Mission Pain and Spine Institute, Mission Viejo, California, USA

**Keywords:** bursitis, corticosteroid, ozone, prolotherapy, shoulder pain, tendinopathy

## Abstract

**Introduction:**

Shoulder tendinopathy is the most common cause of shoulder pain and one of the most common musculoskeletal disorders, for which there is need for better treatment options with fewer complications. This study aimed to determine the effect of local injection of oxygen‐ozone, corticosteroid, and prolotherapy under ultrasound guidance in the treatment of shoulder tendinopathy.

**Methods:**

In this prospective, multicenter, randomized clinical trial, 45 patients with shoulder tendinopathy were selected by a sampling method and randomly assigned to three groups of 15 individuals each (local injection of oxygen‐ozone, corticosteroid, and prolotherapy). Patients’ symptoms were assessed using the Visual Analog Scale (VAS), Constant Score, Shoulder Pain and Disability Index (SPADI), range of motion, and ultrasound findings before treatment, 2 weeks, 2 months, and 6 months after intervention.

**Results:**

VAS scores, SPADI, Constant Score, bursal thickness, and range of motion improved in all three groups following treatment. The lowest pain intensity based on the VAS scale at 2 weeks was observed in the corticosteroid group, while at 2 and 6 months, the lowest pain intensity was observed in the prolotherapy group.

**Conclusion:**

All three treatment options are effective for patients with shoulder tendinopathy. This study demonstrated that the effects of prolotherapy and oxygen‐ozone injections last longer than those of corticosteroid injections. Corticosteroid injections are more effective for short‐term pain relief.

**Trial Registration:** Iranian Registry of Clinical Trials (IRCT): IRCT20151017024572N17

**Key Points**
 This study compared three injection treatments given under ultrasound guidance—corticosteroid, oxygen–ozone, and dextrose prolotherapy—for patients with shoulder tendinopathy in a randomized, multicenter setting.
 Across all groups, patients reported less pain, better shoulder function, greater mobility, and thinner bursae on ultrasound scans.
 Pain reduction happened fastest in the corticosteroid group, especially within the initial 2 weeks after treatment.
 The benefits of prolotherapy and oxygen–ozone were more enduring, with positive effects still noticeable 6 months later.

## 1. Introduction

Shoulder pain is among the most common musculoskeletal conditions, with a monthly prevalence ranging from 18% to 31%, and lifetime prevalence as high as 66.7% [1, 2]. The most common cause of shoulder pain is changes in the periarticular structures of the shoulder joint. These nonbony structures, including ligaments, tendons, and bursae, interact mechanically, and irritation or inflammation in one structure may lead to complications in others [3, 4].

Rotator cuff tendinopathy, which encompasses both tendinitis and tendinosis, is a major source of shoulder pain. While subacromial bursitis may coexist with tendinopathy and contribute to impingement, these conditions can also occur independently without intrinsic tendon involvement. In this study, our primary focus is shoulder tendinopathy, while recognizing that secondary bursa inflammation frequently accompanies the affected tendons [5].

Shoulder tendinopathy and concomitant bursitis are commonly treated with physiotherapy, patient education, home exercises, activity modification, and oral analgesics for moderate pain relief [6]. However, due to the lack of a definitive cure for these conditions and the potential side effects of conventional medications, alternative treatment options have been explored. Among these, minimally invasive interventions like intra‐articular and soft tissue injections, using corticosteroid, oxygen‐ozone, and prolotherapy, have emerged as possible treatment options [7, 8].

For severe shoulder pain management, particularly in cases involving severe tendon and bursa inflammation, corticosteroid injections are proven to reduce pain and inflammation [9, 10]. Oxygen‐ozone, similar to corticosteroids, alleviates pain by inhibiting phospholipase A2 but without the adverse effects typically associated with steroids. Additionally, oxygen‐ozone therapy enhances microcirculation, which is an essential factor in managing chronic pain [11, 12]. By using standardized doses and precise oxygen‐ozone concentration, this treatment induces a transient oxidative state that may safely enhance several beneficial biological responses in the body. These effects include activation of the antioxidant defense system, improved blood flow and oxygen transportation, stimulation of growth factors, and cytokine release [13]. Prolotherapy is a cost‐effective treatment that promotes healing by injecting a solution (commonly dextrose or other agents like phenol and glycerin) into painful ligaments, tendons, and joints [14, 15]. Studies have shown the effectiveness of prolotherapy in treating osteoarthritis, muscle and joint pain, and chronic back pain [15, 16].

Despite the known individual benefits of these interventions, there is a lack of direct comparative studies evaluating their clinical efficacy head‐to‐head. At present, corticosteroid injection remains the most common primary interventional approach. Therefore, the aim of this randomized clinical trial is to address this gap by using corticosteroid injection as the primary comparator against the regenerative effects of oxygen‐ozone and dextrose prolotherapy in the treatment of shoulder tendinopathy.

## 2. Methods

### 2.1. Overview

This interventional, double‐blind clinical trial was conducted on patients with shoulder tendinopathy in accordance with the CONSORT guidelines [17] and approved by the institutional board of Iran University of Medical Sciences (Ethic code number: IR.IUMS.REC 1396.30322, Trial Registry Code: IRCT20151017024572N17). The ethics of this study were in accordance with the Declaration of Helsinki and its later amendments or comparable ethical standards [18]. Forty‐five patients with a primary diagnosis of symptomatic rotator cuff tendinopathy, consistent with our trial registration (IRCT20151017024572N17), were selected in multiple outpatient centers and enrolled in the study after providing informed consent. Concomitant subacromial‐subdeltoid bursitis, frequently secondary to tendinopathy, was recognized and assessed via ultrasound but was not an independent inclusion criterion. Participants were randomly assigned to three groups: local dextrose prolotherapy injection (*n* = 15), local oxygen‐ozone injection (*n* = 15), and local corticosteroid injection (*n* = 15) (Figure 1).

**FIGURE 1:**
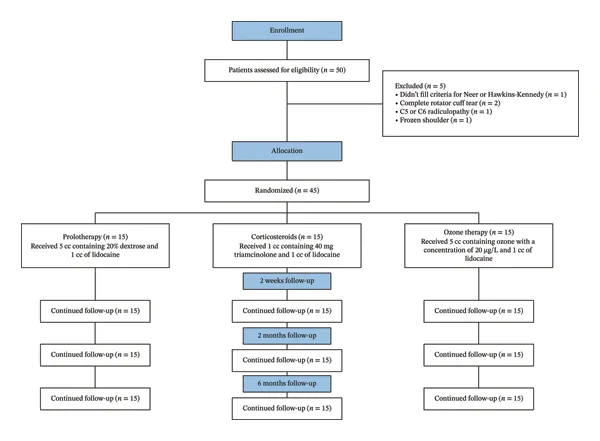
Consort diagram. Subjects were randomized to a 1:1:1 ratio. No patients withdrew from the study after randomization.

Pretreatment demographic data and baseline clinical assessments, including Visual Analog Scale (VAS), Constant Score, Shoulder Pain and Disability Index (SPADI), active range of motion (ROM), and sonographic subacromial bursa thickness, were collected. Same data were collected on follow‐up visits at 2 weeks, 2 months, and 6 months after intervention. All injections were performed under ultrasound guidance, ensuring proper needle placement. In all three groups, patients also participated in exercise therapy, including shoulder ROM exercises, posterior capsule stretching, and isometric exercises of the rotator cuff muscles. The administration of any other medication to the affected shoulder was avoided throughout the course of this study.

### 2.2. Participants

Forty‐five patients with shoulder pain due to rotator cuff tendinopathy were enrolled in this study. All participants met the primary diagnosis of tendinopathy based on positive results from the Neer [19] or Hawkins–Kennedy tests [20], and a VAS score above 4. Sonographic diagnosis of concomitant bursitis was defined as a subacromial bursa thickness exceeding 2 mm or the presence of localized fluid accumulation. Patients with the following conditions were excluded from the study: complete rotator cuff tear, history of upper limb fractures, prior injection in the shoulder joint or bursa within the last 3 months, frozen shoulder, C5 or C6 radiculopathy, and certain underlying conditions such as diabetes, coagulopathy, rheumatological disorders, infectious, and tumors.

### 2.3. Intervention

After prepping the affected shoulder in a sterile fashion, under ultrasound guidance using a lateral to medial approach, 1 cc lidocaine 1% was injected to anesthetize the skin and subcutaneous path to the subacromial bursa. Then, one of the following injections was administered to each participant under ultrasound guidance.

The lateral shoulder was prepared with antiseptic solution. A sterile‐covered linear ultrasound probe was placed longitudinally, and a 23‐gauge needle was advanced into the subacromial bursa under ultrasound guidance using an in‐plane, lateral‐to‐medial approach.

In the prolotherapy group (PrG), 5 cc of 20% dextrose; in the oxygen‐ozone group (OzG), 5 cc of ozone with a concentration of 20 μg/L; and in the corticosteroid group (CoG), 1 cc of triamcinolone 40 mg/mL.

All injections were performed by a physical medicine and rehabilitation specialist with fellowship training in musculoskeletal ultrasound and fluoroscopic guided interventions.

### 2.4. Randomization and Blinding

This clinical trial employed random sequence generation using RAS software with a block randomization method (using a fixed block size) to ensure an equal 1:1:1 allocation ratio among the three groups. To ensure strict allocation concealment prior to participant enrollment, sequentially numbered, opaque, sealed envelopes containing the group assignments were prepared by an independent coordinator. These envelopes were opened only after the patient met all eligibility criteria and provided informed consent, immediately prior to the intervention.

Due to the distinct physical properties of the injected preparations (gas vs. liquids), the injecting physician could not be blinded to the treatment allocation. However, strict blinding was maintained for all other involved parties in line with CONSORT recommendations. Participants remained completely unaware of their group assignment. Furthermore, the clinical outcome assessors evaluating the patients during follow‐ups, and the data analysts, were entirely blinded to the group allocations. To support patient blinding, all syringes were prepared by independent medical personnel, covered with sterile gauze, and labeled strictly with the participant’s identification number.

### 2.5. Outcome Measures

1. Demographic Information: A questionnaire was collected before treatment, including, age, gender, weight, height, marital status, education level, occupation, medical history, medication history, and duration of symptoms related to their shoulder pain.
2. Clinical Global Impression (CGI): A questionnaire measures overall clinical perception. It is a two‐item instrument that assesses overall improvement (CGI‐I) and treatment effectiveness (CGI‐E) on a seven‐point Likert scale [21].
3. Patient Global Impression of Improvement (PGI‐I): A single‐item assessment of the patient’s overall improvement. Patients completed a questionnaire on a seven‐point Likert scale [22].
4. VAS: This is a visual tool for measuring the intensity of pain on a scale of 0–10 with pain classified as mild (1–3), moderate (4–6), or severe (7–10) [23].
5. SPAD: It assesses pain and disability related to shoulder conditions. SPADI includes two variables: pain intensity and disability. Pain may range from 0 to 50 and disability from 0 to 80. The final score is the sum of the two variables and may range between 0 and 130 [24].
6. Constant Shoulder Score: This questionnaire assesses shoulder function based on subjective and objective variables. The two subjective variables include pain (15 points) and ADL (activity level 10 points and arm movement 10 points). The two objective variables include arm abduction strength (25 points) and shoulder joint ROM (40 points), all measured by the clinician. The final score is calculated when the sum of all variable points is subtracted from 100 [25].
7. Active ROM: A clinician measures active abduction and forward flexion in degrees using a goniometer.
8. Subacromial Bursa Thickness: Measured by specialists in millimeters under ultrasound guidance.

### 2.6. Statistical Analysis

The following formula was used to calculate the sample size:

(1) n=2×Z12−α/+Z1−β2×σ2Δ2,

where *n* is the required sample size for each group; *σ* is the standard deviation of the outcome (pain severity or functional disability) in each group based on recent studies [26], which is assumed to be approximately 1.8; *Z*_1−*α*/2_ is the *Z*‐score for the desired significance level (approximately 1.96 for a two‐sided test with *α* = 0.05); *Z*_1−*β*_ is the *Z*‐score for the power of the test (approximately 1.04 for 85% power); and Δ is the expected mean difference between the two groups (effect size), which is assumed to be approximately 2.

The initial calculation yielded a required sample size of 13 participants per group. Considering a 15% dropout rate, the final calculation adjusted the sample size to 15 participants per group.

After completing the data collection, the data were analyzed using version 24 of SPSS software. In order to report descriptive criteria, central indices (such as mean) and dispersion (standard deviation and range of changes) were used to describe quantitative variables. Frequency and percentage were used to report qualitative variables.

The data distribution was examined using the Kolmogorov–Smirnov (K‐S) test. To evaluate the trajectory of clinical outcomes within each treatment group during the follow‐up periods (within‐group analyses presented in Table 1), a repeated measures ANOVA was utilized for normally distributed variables, followed by Bonferroni post hoc tests for pairwise comparisons. For nonparametric data, the Friedman test was applied, followed by post hoc Wilcoxon signed‐rank tests. To examine the interaction effect of time and group, and to perform between‐group pairwise comparisons at specific follow‐up intervals (Table 2), repeated measures ANOVA with Bonferroni correction was employed. In the case of baseline values that had a significant difference, the baseline value was entered as a covariate. The results were interpreted based on the significance level of *p* < 0.05.

**TABLE 1:** Within‐group comparisons of clinical outcomes across short‐term, mid‐term, and long‐term follow‐up intervals.

Outcomes & time points	Prolotherapy group (PrG)	Ozone group (OzG)	Corticosteroid group (CoG)
VAS score			
Baseline	7.60 ± 1.40	7.87 ± 1.06	7.33 ± 1.39
2 weeks	4.20 ± 1.78	5.47 ± 1.30	2.93 ± 0.88
2 months	2.93 ± 2.01	4.13 ± 1.06	2.73 ± 1.38
6 months	2.73 ± 1.48	4.20 ± 1.61	3.73 ± 1.53
Short‐term effect P1 (Base vs 2 w)	3.40 (2.31–4.48), *p* < 0.001	2.40 (1.31–3.48), *p* < 0.001	4.40 (3.31–5.48), *p* < 0.001
Mid‐term effect P2 (Base vs 2 m)	4.66 (3.33–5.99), *p* < 0.001	3.73 (2.40–5.06), *p* < 0.001	4.60 (3.26–5.93), *p* < 0.001
Long‐term effect P3 (Base vs 6 m)	4.86 (3.60–6.12), *p* < 0.001	3.66 (2.40–4.92), *p* < 0.001	3.60 (2.33–4.86), *p* < 0.001
Constant shoulder score			
Baseline	62.60 ± 12.37	70.33 ± 11.44	66.80 ± 7.30
2 weeks	37.67 ± 11.62	58.13 ± 13.72	31.60 ± 11.76
2 months	32.00 ± 12.05	50.53 ± 12.79	31.33 ± 14.61
6 months	27.60 ± 10.19	46.93 ± 13.47	39.93 ± 12.44
Short‐term effect P1 (Base vs 2 w)	24.93 (15.84–34.02), *p* < 0.001	12.20 (3.11–21.28), *p* = 0.004	35.20 (26.11–44.28), *p* < 0.001
Mid‐term effect P2 (Base vs 2 m)	30.60 (20.66–40.53), *p* < 0.001	19.80 (9.86–29.73), *p* < 0.001	35.46 (25.53–45.40), *p* < 0.001
Long‐term effect P3 (Base vs 6 m)	35.00 (26.05–43.94), *p* < 0.001	23.40 (14.45–32.34), *p* < 0.001	26.86 (17.92–35.80), *p* < 0.001
Spadi score			
Baseline	91.20 ± 18.70	82.60 ± 14.47	78.20 ± 13.08
2 weeks	54.60 ± 23.15	64.80 ± 17.40	43.40 ± 17.47
2 months	38.13 ± 26.21	60.20 ± 17.44	34.53 ± 21.13
6 months	36.00 ± 20.84	58.80 ± 15.32	37.67 ± 11.33
Short‐term effect P1 (Base vs 2 w)	36.60 (23.80–49.39), *p* < 0.001	17.80 (5.00–30.59), *p* = 0.002	34.80 (22.00–47.59), *p* < 0.001
Mid‐term effect P2 (Base vs 2 m)	53.06 (37.48–68.64), *p* < 0.001	22.40 (6.82–37.97), *p* = 0.002	43.66 (28.08–59.24), *p* < 0.001
Long‐term effect P3 (Base vs 6 m)	55.20 (40.84–69.55), *p* < 0.001	23.80 (9.44–38.15), *p* < 0.001	40.53 (26.17–54.88), *p* < 0.001
Shoulder abduction (degrees)			
Baseline	104.33 ± 16.78	93.33 ± 27.94	113.00 ± 19.43
2 weeks	122.67 ± 20.43	109.67 ± 29.84	125.67 ± 18.88
2 months	126.33 ± 19.95	114.33 ± 31.50	120.33 ± 17.05
6 months	125.00 ± 18.02	117.00 ± 28.95	119.67 ± 14.20
Short‐term effect P1 (Base vs 2 w)	18.33 (10.15–26.51), *p* < 0.001	16.33 (8.15–24.51), *p* < 0.001	12.67 (4.48–20.84), *p* = 0.001
Mid‐term effect P2 (Base vs 2 m)	22.00 (9.86–34.13), *p* < 0.001	21.00 (8.86–33.13), *p* < 0.001	7.33 (−4.79–19.46), *p* = 0.610
Long‐term effect P3 (Base vs 6 m)	20.66 (8.48–32.85), *p* < 0.001	23.66 (11.48–35.85), *p* < 0.001	6.66 (−5.51–18.85), *p* = 0.824
Shoulder forward flexion (degrees)			
Baseline	107.00 ± 18.59	104.07 ± 23.22	113.67 ± 21.66
2 weeks	124.00 ± 22.61	132.00 ± 28.46	130.00 ± 19.08
2 months	130.00 ± 24.64	134.67 ± 25.52	133.00 ± 15.67
6 months	130.00 ± 24.12	135.67 ± 24.11	132.67 ± 12.79
Short‐term effect P1 (Base vs 2 w)	17.00 (3.90–30.10), *p* = 0.005	27.93 (14.83–41.03), *p* < 0.001	16.33 (3.23–29.43), *p* = 0.008
Mid‐term effect P2 (Base vs 2 m)	23.00 (9.75–36.24), *p* < 0.001	30.60 (17.35–43.84), *p* < 0.001	19.33 (6.09–32.57), *p* = 0.001
Long‐term effect P3 (Base vs 6 m)	23.00 (9.46–36.53), *p* < 0.001	31.60 (18.06–45.13), *p* < 0.001	19.00 (5.46–32.53), *p* = 0.002
Subacromial bursa thickness (mm)			
Baseline	1.55 ± 0.19	1.81 ± 0.39	1.83 ± 0.43
2 weeks	1.18 ± 0.23	1.22 ± 0.29	1.01 ± 0.45
2 months	1.06 ± 0.25	1.14 ± 0.32	0.93 ± 0.43
6 months	0.94 ± 0.14	1.08 ± 0.31	1.03 ± 0.45
Short‐term effect P1 (Base vs 2 w)	0.36 (0.17–0.56), *p* < 0.001	0.59 (0.40–0.79), *p* < 0.001	0.81 (0.61–1.01), *p* < 0.001
Mid‐term effect P2 (Base vs 2 m)	0.48 (0.28–0.68), *p* < 0.001	0.67 (0.47–0.87), *p* < 0.001	0.90 (0.70–1.10), *p* < 0.001
Long‐term effect P3 (Base vs 6 m)	0.61 (0.41–0.80), *p* < 0.001	0.73 (0.53–0.92), *p* < 0.001	0.80 (0.60–0.99), *p* < 0.001

*Note:* Data are reported as mean ± SD for time points, and mean difference (95% CI) for treatment effects.

**TABLE 2:** Between‐group pairwise comparisons for clinical outcomes at each follow‐up interval.

Outcomes & time points	PrG vs. OzG	PrG vs. CoG	OzG vs. CoG
	MD (95% CI), *p* value	MD (95% CI), *p* value	MD (95% CI), *p* value
VAS score			
Short‐term (2 weeks)	−1.27 (−2.44 to −0.10), *p* = 0.102	1.27 (0.23–2.31), *p* = 0.057	2.54 (1.72–3.36), *p* < 0.001
Mid‐term (2 months)	−1.20 (−2.39 to −0.01), *p* = 0.144	0.20 (−1.09–1.49), *p* > 0.99	1.40 (0.48–2.32), *p* = 0.012
Long‐term (6 months)	−1.47 (−2.62 to −0.32), *p* = 0.042	−1.00 (−2.13 to 0.13), *p* = 0.240	0.47 (−0.70–1.64), *p* > 0.99
Constant score			
Short‐term (2 weeks)	−20.46 (−29.96 to −10.96), *p* < 0.001	6.07 (−2.68–14.82), *p* = 0.490	26.53 (16.97–36.09), *p* < 0.001
Mid‐term (2 months)	−18.53 (−27.83 to −9.23), *p* = 0.001	0.67 (−9.34–10.68), *p* > 0.99	19.20 (8.94–29.46), *p* = 0.002
Long‐term (6 months)	−19.33 (−28.26 to −10.40), *p* < 0.001	−12.33 (−20.83 to −3.83), *p* = 0.018	7.00 (−2.69–16.69), *p* = 0.450
SPADI score			
Short‐term (2 weeks)	−10.20 (−25.51 to 5.11), *p* = 0.550	11.20 (−4.14–26.54), *p* = 0.440	21.40 (8.37–34.43), *p* = 0.006
Mid‐term (2 months)	−22.07 (−38.72 to −5.42), *p* = 0.033	3.60 (−14.20–21.40), *p* > 0.99	25.67 (11.19–40.15), *p* = 0.003
Long‐term (6 months)	−22.80 (−36.46 to −9.14), *p* = 0.006	−1.67 (−14.20 to 10.86), *p* > 0.99	21.13 (11.05–31.21), *p* < 0.001
Shoulder abduction			
Short‐term (2 weeks)	13.00 (−6.13–32.13), *p* = 0.520	−3.00 (−17.70 to 11.70), *p* > 0.99	−16.00 (−34.68 to 2.68), *p* = 0.270
Mid‐term (2 months)	12.00 (−7.70–31.70), *p* = 0.660	6.00 (−7.86–19.86), *p* > 0.99	−6.00 (−24.94 to 12.94), *p* > 0.99
Long‐term (6 months)	8.00 (−10.02–26.02), *p* > 0.99	5.33 (−6.79–17.45), *p* > 0.99	−2.67 (−19.71 to 14.37), *p* > 0.99
Subacromial bursa			
Short‐term (2 weeks)	−0.04 (−0.22 to 0.14), *p* > 0.99	0.17 (−0.10–0.44), *p* > 0.99	0.21 (−0.08–0.50), *p* = 0.450
Mid‐term (2 months)	−0.08 (−0.28 to 0.12), *p* > 0.99	0.13 (−0.14–0.40), *p* > 0.99	0.21 (−0.08–0.50), *p* = 0.450
Long‐term (6 months)	−0.14 (−0.32 to 0.04), *p* = 0.360	−0.09 (−0.34 to 0.16), *p* > 0.99	0.05 (−0.24–0.34), *p* > 0.99

## 3. Results

### 3.1. Demographic

A total of 45 patients were randomly assigned to three groups: PrG (*n* = 15), OzG (*n* = 15), and CoG (*n* = 15). There were no significant differences between the groups regarding gender distribution and all other baseline characteristics (Table 3).

**TABLE 3:** Demographic and baseline characteristics of patients before initiation of treatment.

Parameters		PrG		OzG		CoG		*p* values
		Number/mean	Percentage/SD	Number/mean	Percentage/SD	Number/mean	Percentage/SD	
Gender	Male	4	26.67%	2	13.33%	6	40%	0.269
	Female	11	73.33%	13	86.67%	9	60%	

Age (year)		62.13	10.25	59.40	10.30	55.8	14.71	0.355

Duration of symptoms (months)		9.53	7.41	10.80	9.53	7.60	4.80	0.506

VAS Score		7.60	1.40	7.87	1.06	7.33	1.39	0.535

Constant Shoulder Score		62.60	12.37	70.33	11.44	66.80	7.30	0.148

SPADI		91.20	18.70	82.60	14.47	78.20	13.083	0.079

Subacromial bursa thickness (mm)		1.553	0.192	1.819	0.395	1.833	0.435	0.065

Supraspinatus tendon thickness (mm)		5.740	0.258	5.619	1.217	5.587	0.958	0.889

Acromiohumeral interval (mm)		10.973	1.035	11.204	2.045	11.058	1.819	0.931

Abduction (degrees)		104.33	16.78	93.33	27.94	113.00	19.43	0.059

Forward flexion (degrees)		107.00	18.59	104.07	23.22	113.67	21.66	0.454

*Note:* PrG, prolotherapy group; OzG, ozone group; CoG, corticosteroid group.

Abbreviations: SD, standard deviation; SPADI, Shoulder Pain and Disability Index.

### 3.2. VAS

Pain scores (VAS) dropped significantly across all groups during the 6‐month study. Within‐group testing showed these improvements occurred at every follow‐up compared to baseline ($*p* < 0.001$, Table 2). The timeline of recovery, however, relied heavily on the specific intervention. A significant time‐by‐group interaction was observed (*p* = 0.001), meaning the therapies differed clearly in onset and duration of relief (Table 3).

Corticosteroids delivered the fastest pain control. At 2 weeks, the CoG cohort showed a marked superiority over the OzG group (MD: 2.54, 95% CI: 1.72 to 3.36, *p* < 0.001). Yet, this acute advantage faded. By the 6‐month mark, regenerative treatments took the lead. Prolotherapy offered the strongest long‐term pain reduction, proving statistically superior to ozone therapy at the final assessment (MD: −1.47, 95% CI: −2.62 to −0.32, *p* = 0.042).

### 3.3. Shoulder Function and Disability (SPADI and Constant Score)

Functional recovery followed a trajectory much like the pain scores. Both the Constant and SPADI tools revealed a significant time‐by‐group interaction (*p* < 0.001). Every treatment group achieved measurable functional gains over the 6 months (Table 2), but the pacing of these improvements differed considerably across the therapies (Table 3).

Corticosteroids drove an immediate improvement in shoulder function. At the 2‐week mark, patients in the CoG had significantly better Constant scores than those receiving ozone (MD: 26.53, 95% CI: 16.97 to 36.09, *p* < 0.001). This early advantage, however, did not hold. Prolotherapy ultimately delivered the most resilient functional outcomes. By 6 months, the PrG outperformed the CoG on the Constant score (MD: −12.33, 95% CI: −20.83 to −3.83, *p* = 0.018). It also proved statistically superior to ozone therapy in lowering SPADI disability scores at the final visit (MD: −22.80, 95% CI: −36.46 to −9.14, *p* = 0.006).

### 3.4. ROM

Treatment type heavily dictated the recovery of shoulder abduction. The time‐by‐group interaction was significant (*p* = 0.018, Table 3), highlighting distinct patterns of improvement.

Looking at within‐group changes (Table 2), corticosteroids provided a strictly short‐lived boost. Patients in the CoG gained significant abduction at 2 weeks (MD: 12.67, 95% CI: 4.48 to 20.84, *p* = 0.001). After that point, their progress stalled. These improvements lost statistical significance at the two‐ and 6‐month evaluations (*p* = 0.610 and *p* = 0.824, respectively). The OzG and PrG followed a different path. Both cohorts maintained steady, significant gains in abduction from baseline right through to the final follow‐up (*p* < 0.001). Even with these distinct recovery timelines, no single treatment emerged as statistically superior to the others in direct between‐group testing at 6 months.

Forward flexion showed a much more uniform response. The time‐by‐group interaction failed to reach significance (*p* = 0.207), indicating all three therapies produced a similar pace of recovery. Within‐group testing confirmed this shared trend; patients in every group achieved and sustained significant gains in forward flexion from baseline up to the final assessment (Table 2).

### 3.5. Subacromial Bursa Thickness

Ultrasound tracking of the subacromial bursa thickness revealed a highly significant time‐by‐group interaction (*p* < 0.001). The specific injection heavily influenced the timeline of structural healing.

Looking at the groups individually (Table 2), every cohort saw a steady and significant drop in bursa thickness across all follow‐up visits (*p* < 0.001). All three therapies effectively reduced the structural inflammation over the study period. Direct between‐group comparisons, however, leveled the playing field. Bonferroni‐adjusted tests (Table 3) found no statistical differences among the therapies at any specific time point (*p* > 0.05). The early pace of bursa reduction may differ, but the final structural outcomes end up being clinically comparable regardless of the injection used.

### 3.6. Overall Clinical Improvement

Global clinical efficacy and patient‐perceived recovery were tracked using the CGI‐I, CGI‐E, and PGI‐I scales. When comparing the treatments directly, Kruskal–Wallis testing found no significant differences among the groups at any follow‐up point (*p* > 0.05). The choice of injection did not statistically alter the overall global impression scores.

Within each group, however, the clinical progress was clear. Wilcoxon signed‐rank tests confirmed that patients across all three cohorts experienced significant, ongoing improvements from the 2‐week evaluation to both the 2‐month and 6‐month marks (*p* < 0.05).

## 4. Discussion

### 4.1. Pain Reduction, Overall and Functional Improvement

This study assessed the effectiveness of three different injection therapies—corticosteroid, oxygen‐ozone, and prolotherapy—in managing shoulder tendinopathy. Our results demonstrated that all three treatments contributed to pain reduction as measured by VAS, SPADI, and Constant score, as well as overall (CGI‐E, CGI‐I, and PGI‐I) and functional improvement. However, the magnitude and durability of these effects varied over time.

Corticosteroid injections showed the most rapid pain relief in the short term, particularly within the first 2 weeks after treatment, as supported by our short‐term post hoc analyses. Corticosteroids alleviate pain primarily by suppressing inflammation [27, 28]. Their mechanism of action is to inhibit phospholipase A2, therefore lowering the synthesis of inflammatory mediators such as prostaglandins and leukotrienes. This results in reduced swelling, decreased tissue irritability, and fewer nociceptive signals [29]. Furthermore, immunosuppressive effects of corticosteroids occur by preventing leukocytes migration and inhibition of pro‐inflammatory cytokines such as TNF‐*α*, IL‐1, and IL‐6. This lessens tissue damage and nociceptor sensitization [30]. They also stabilize neural membranes and modulate ion channels, reducing nerve excitability and pain signal transmission [31]. In our study, the efficacy of steroid injection diminished over time, with many patients experiencing symptom recurrence at the 6‐month follow‐up visit. This decline in efficacy has been widely reported in the literature [7, 32–34], with concerns regarding the potential side effects such as cartilage degeneration, tendon weakening, and increased risk of rupture with repeated corticosteroid injections [33]. Additionally, corticosteroids masking the underlying pathology without directly promoting tendon repair [35].

On the other hand, prolotherapy and oxygen‐ozone injections demonstrated more durable, long‐term benefits. Prolotherapy utilizes dextrose to induce a localized inflammatory response that promotes tissue healing and alleviates pain [36, 37]. The hypertonic dextrose solution triggers osmotic stress causing localized cell lysis and release of pro‐inflammatory mediators, including transformational growth factor‐beta (TGF‐β) and connective tissue growth factor (CTGF). This inflammatory process stimulates fibroblast proliferation leading to formation and deposition of new collagen, thereby strengthening tendon and ligament structures [38, 39]. Moreover, hypertonic dextrose increases angiogenesis, promoting the repair of injured tendon fibers [40]. Unlike corticosteroids, prolotherapy requires several weeks to months to demonstrate notable changes as it relies on endogenous healing processes [8]. Prolotherapy may be less appealing for patients seeking immediate relief.

Similarly, oxygen‐ozone therapy provided long‐term symptom relief. As an emerging intervention for treatment of shoulder tendinopathies, oxygen‐ozone therapy provides anti‐inflammatory, analgesic, and regenerative effects. It reduces pro‐inflammatory cytokines, including TNF‐α, IL‐1β, and IL‐6, and promotes release of anti‐inflammatory cytokines like IL‐4, IL‐10, and TGF‐β [41]. Furthermore, by improving microcirculation and oxygenation through nitric oxide (NO)‐mediated vasodilation, oxygen‐ozone therapy accelerates tissue regeneration [42]. These regenerative effects of oxygen‐ozone and dextrose prolotherapy could explain the delayed onset but longer‐lasting pain reduction observed in our patients, contrasting with the rapid yet transient relief from corticosteroid injections.

Our findings are consistent with existing literature concerning the comparative efficacy of corticosteroid, prolotherapy, and ozone injections. The transient benefits of corticosteroids have been extensively documented, with studies reporting a decline in therapeutic effects beyond the acute phase of treatment [33, 34, 43, 44]. In contrast, prolotherapy is associated with regenerative tissue responses that contributes to sustained clinical improvement [36–40], while ozone therapy exhibits both anti‐inflammatory properties and circulatory enhancement, supporting its role as an intermediate‐duration relief [41, 42].

Sabaah et al. demonstrated the superiority of prolotherapy over corticosteroids and platelet‐rich plasma in patients with rotator cuff tendinopathy after a 3‐month follow‐up, supporting the 6‐month efficacy observed in our study. Similarly, Raeissadat et al. reported that although ozone therapy was initially effective in alleviating knee osteoarthritis symptoms, its therapeutic effects diminished by the 6‐month mark, consistent with the intermediate outcomes observed in our study [14, 44]. Furthermore, Babaei‐Ghazani et al. observed that both prolotherapy and ozone yielded superior outcomes compared to corticosteroids in managing pes anserine bursitis over an 8‐week period, reinforcing the longer‐lasting utility of these interventions [45].

Our results demonstrated significant improvements in shoulder ROM across all treatment groups. This can be attributed to reduced inflammation and pain in all our treatment methods [43, 45, 46]. Additionally, regenerative properties of prolotherapy and oxygen‐ozone could enhance shoulder function. However, the long‐term impact of corticosteroids on tendon structure remains a concern [47]. Forward flexion improved uniformly across the board. Pairwise testing confirmed that patients in all groups gained significant mobility, with no single therapy outperforming the rest.

The abduction results, however, told a very different story. Ozone and prolotherapy drove continuous, significant improvements right through to the final follow‐up. Corticosteroid benefits peaked early. The initial gains in abduction for the corticosteroid cohort quickly stalled, losing statistical significance after just 2 weeks. Ultimately, for restoring and maintaining long‐term shoulder mobility, regenerative options like ozone and prolotherapy clearly provide a more durable advantage over steroids.

Our sonographic findings revealed a significant longitudinal decrease in subacromial bursa thickness within all treatment groups, which aligns with prior studies [34, 48–50]. Although between‐group comparisons did not identify one single agent as statistically superior to the others, the rapid reduction in subacromial bursa thickness following corticosteroid injection can be attributed to its potent anti‐inflammatory effects, suppressing cytokine activity (IL‐1, TNF‐α) and reducing local fluid accumulation [47]. The hypertonic dextrose injection (prolotherapy) induces a controlled inflammatory response, leading to fibroblast activation and extracellular matrix remodeling. Over time, this restores normal bursa function and reduces excessive swelling, contributing to the progressive reduction in bursa thickness [39]. Oxygen‐ozone therapy reduces chronic inflammation and oxidative stress in the bursa by promoting antioxidant activity and cytokine modulation [41]. This results in gradual and sustained reduction of subacromial bursa thickness observed at 6 months.

To the best of our knowledge, this is the first multicenter randomized clinical trial directly comparing corticosteroid, oxygen–ozone, and dextrose prolotherapy under ultrasound guidance for shoulder tendinopathy. Our results provide new clinical insight by showing that although corticosteroids offer superior short‐term pain relief, oxygen–ozone and prolotherapy are associated with more sustained improvements in pain and function at 6 months. These findings support the role of regenerative injection therapies as evidence‐based alternatives for longer‐term conservative management.

### 4.2. Strengths and Limitations

The key strength of this trial is its randomized, multicenter, double‐blind design, which minimizes bias and improves reliability of outcomes. The use of ultrasound guidance ensured precise injection placement and reducing treatment variability. Additionally, including several therapeutic modalities made it possible to compare their efficacy, therefore offering important information for clinical decision‐making.

Nonetheless, this study has limitations. Post‐treatment activity levels which could influence outcomes were not controlled. The relatively small sample size may also limit the generalizability of the findings. While a 6‐month follow‐up period offered valuable information on prolonged effects, longer‐term studies are necessary to assess the durability of these interventions over 12 months or more. Future research utilizing imaging techniques could provide a more comprehensive understanding of the regenerative effects of prolotherapy and oxygen‐ozone therapy.

## 5. Conclusion

This study demonstrated that while corticosteroid injections provide rapid, short‐term pain relief, their long‐term efficacy is inferior to dextrose prolotherapy and oxygen‐ozone therapy. Prolotherapy appears to offer sustained pain relief and functional improvement by promoting tissue regeneration. Oxygen‐ozone therapy, with its anti‐inflammatory and microcirculatory‐enhancing properties, also provides promising long‐term benefits with minimal side effects. The choice of treatment should consider both short‐term and long‐term therapeutic goals based on individual patient needs.

## Author Contributions

Conceptualization: Arash Babaei‐Ghazani and Farzane Rouzegari; data collection, analysis, and visualization: Arash Babaei‐Ghazani, Farzane Rouzegari, Naseh Yousefi, Lobaneh Janbazi, and Hosna Soleymanzadeh; writing–original draft: Arash Babaei‐Ghazani, Farzane Rouzegari, Naseh Yousefi, Lobaneh Janbazi, Hosna Soleymanzadeh; writing–review and editing: Arash Babaei‐Ghazani, Farzane Rouzegari, Naseh Yousefi, Lobaneh Janbazi, Hosna Soleymanzadeh; resources: Bijan Forogh; supervision: Arash Babaei‐Ghazani, and Bijan Forogh.

## Funding

This study had no funding support and received no grants.

## Disclosure

All authors have read and approved the manuscript prior to submission.

## Ethics Statement

We conducted this interventional double‐blind clinical trial entitled “The effect of local injection of ozone, corticosteroid and prothorectropy under ultrasound guidance in the treatment of inflammation of shoulder tendons: a randomized, prospective, multicenter double blind clinical trial with positive control group” after the institutional board review by Iran University of medical sciences (Ethics code number: IR.IUMS.REC 1396.30322, July 2017). The ethics of this study are in accordance with the Declaration of Helsinki and its later amendments or comparable ethical standards. Trial Registry Code: IRCT20151017024572N17, July 2018.

## Consent

Written informed consent was obtained from the patient for publication and any accompanying images.

## Conflicts of Interest

The authors declare no conflicts of interest.

## Data Availability

The data that support the findings of this study are available upon request from the corresponding author. The data are not publicly available due to privacy or ethical restrictions.

## References

[1] Thompson M. K. , Axford J. S. , and Sofat N. , Rheumatoid Arthritis in 2011: An Editorial, Best Practice & Research Clinical Rheumatology. (2011) 25, no. 4, 435–446, 10.1016/j.berh.2011.10.011. 22137916

[2] Zhang Q. , Chen D. , Shen Y. , Bian M. , Wang P. , and Li J. , Incidence and Prevalence of Poststroke Shoulder Pain Among Different Regions of the World: A Systematic Review and Meta-Analysis, Frontiers in Neurology. (2021) 12, 10.3389/fneur.2021.724281. PMC8600331 34803873

[3] Muto M. , Andreula C. , and Leonardi M. , Treatment of Herniated Lumbar Disc by Intradiscal and Intraforaminal Oxygen-Ozone (O2-O3) Injection, Journal of Neuroradiology. (2004) 31, no. 3, 183–189. 10.1016/s0150-9861(04)96989-1 15356443

[4] Uhthoff H. and Sarkar K. , Periarticular Soft Tissue Conditions Causing Pain in the Shoulder, Current Opinion in Rheumatology. (1992) 4, no. 2, 241–246, 10.1097/00002281-199204000-00018. 1581153

[5] Faruqi T. and Rizvi T. J. , Subacromial Bursitis, StatPearls, 2019, StatPearls Publishing, Treasure Island, FL, USA. 31082140

[6] Sutaria R. and Sutaria R. , Subacromial Bursitis and Impingement, StatPearls, 2017, StatPearls Publishing, 51–54.

[7] Babaei-Ghazani A. , Eftekharsadat B. , Soleymanzadeh H. , and ZoghAli M. , Ultrasound-Guided Pes Anserine Bursitis Injection Choices: Prolotherapy or Oxygen-Ozone or Corticosteroid: A Randomized Multicenter Clinical Trial, American Journal of Physical Medicine & Rehabilitation. (2024) 103, no. 4, 310–317, 10.1097/phm.0000000000002343. 37752656

[8] Nasiri A. , Jahromi L. S. M. , Vafaei M. A. , Parvin R. , Fakheri M. , and Sadeghi S. , Comparison of the Effectiveness of Ultrasound-Guided Prolotherapy in Supraspinatus Tendon With Ultrasound-Guided Corticosteroid Injection of Subacromial Subdeltoid Bursa in Rotator Cuff-Related Shoulder Pain: A Clinical Trial Study, Advanced Biomedical Research. (2021) 10. 10.4103/abr.abr_181_20 PMC8204815 34195156

[9] Chae J. and Jedlicka L. , Subacromial Corticosteroid Injection for Poststroke Shoulder Pain: An Exploratory Prospective Case Series, Archives of Physical Medicine and Rehabilitation. (2009) 90, no. 3, 501–506, 10.1016/j.apmr.2008.10.011. PMC4193293 19254618

[10] Hsieh L. , Lin Y.-J. , Hsu W. et al., Comparison of the Corticosteroid Injection and Hyaluronate in the Treatment of Chronic Subacromial Bursitis: A Randomized Controlled Trial, Clinical Rehabilitation. (2021) 35, no. 9, 1305–1316, 10.1177/02692155211007799. 33858205

[11] Borrelli E. , Mechanism of Action of Oxygen Ozone Therapy in the Treatment of Disc Herniation and Low Back Pain, Acta Neurochirurgica Supplement. (2011) 108, 123–125. 10.1007/978-3-211-99370-5_19 21107948

[12] Wang M.-Y. , Xing C. , Wang J. N. , Yi L. , Lin X. W. , and Fu Z. J. , Therapeutic Dosage of Ozone Inhibits Autophagy and Apoptosis of Nerve Roots in a Chemically Induced Radiculoneuritis Rat Model, European Review for Medical and Pharmacological Sciences. (2018) 22, no. 6, 1787–1797. 10.26355/eurrev_201803_14598 29630128

[13] Bocci V. A. , Scientific and Medical Aspects of Ozone Therapy. State of the Art, Archives of Medical Research. (2006) 37, no. 4, 425–435, 10.1016/j.arcmed.2005.08.006. 16624639

[14] Raeissadat S. A. , Tabibian E. , Rayegani S. M. , Rahimi-Dehgolan S. , and Babaei-Ghazani A. , An Investigation Into the Efficacy of Intra-Articular Ozone (O(2)-O(3)) Injection in Patients With Knee Osteoarthritis: A Systematic Review and Meta-Analysis, Journal of Pain Research. (2018) 11, 2537–2550, 10.2147/jpr.s175441. PMC6207244 30498370

[15] Taheem Y. and Suvar T. , Prolotherapy: Review With Background History, Mechanism of Action, and Current Evidence, 2022, ASRA News.

[16] Bae G. , Kim S. , Lee S. , Lee W. , and Lim Y. , Prolotherapy for the Patients With Chronic Musculoskeletal Pain: Systematic Review and Meta-Analysis, Anesthesia and Pain Medicine. (2020) 16, no. 1, 81–95, 10.17085/apm.20078. PMC7861898 33348947

[17] Bennett J. A. , The Consolidated Standards of Reporting Trials (CONSORT): Guidelines for Reporting Randomized Trials, Nursing Research. (2005) 54, no. 2, 128–132, 10.1097/00006199-200503000-00007. 15778654

[18] World Medical Association , World Medical Association Declaration of Helsinki: Ethical Principles for Medical Research Involving Human Subjects, JAMA. (2013) 310, no. 20, 2191–2194. 10.1001/jama.2013.281053 24141714

[19] Neer C. S. , Impingement Lesions, Clinical Orthopaedics and Related Research. (1983) 173, 70–77, 10.1097/00003086-198303000-00010. 6825348

[20] Hawkins R. J. and Kennedy J. C. , Impingement Syndrome in Athletes, The American Journal of Sports Medicine. (1980) 8, no. 3, 151–158, 10.1177/036354658000800302. 7377445

[21] Omranifard V. , Abounoori M. , Babakhanian M. et al., Translation and Psychometric Properties of the Persian Version of the Health of the Nation Outcome Scales for Elderly People (Honos65+), Aging Medicine. (2021) 4, no. 2, 135–145, 10.1002/agm2.12153. PMC8251871 34250432

[22] Guy W. , ECDEU Assessment Manual for Psychopharmacology, 1976, US Department of Health, Education, and Welfare, Rockville, MD.

[23] Akbari H. , Ghasemi M. , Yegani T. et al., Million Visual Analogue Scale Questionnaire: Validation of the Persian Version, Asian Spine Journal. (2019) 13, no. 2, 242–247, 10.31616/asj.2018.0223. PMC6454270 30481975

[24] Ebrahimzadeh M. , Birjandinejad A. , Golhasani F. , Moradi A. , Vahedi E. , and Kachooei A. , Cross-Cultural Adaptation, Validation, and Reliability Testing of the Shoulder Pain and Disability Index in the Persian Population With Shoulder Problems, International Journal of Rehabilitation Research. (2014) 38, no. 1, 84–87, 10.1097/mrr.0000000000000088. 25305009

[25] Amroodi M. N. , Mostafavi K. , Sobhany A. et al., Translation and Cultural Adaptation of Constant Score Into Persian, Journal of Iranian Medical Council. (2023) 10.18502/jimc.v6i4.13447.

[26] Sharareh R. and Fatemeh Doroodi D. , Comparing the Effects of Simultaneous Intra-Articular and Periarticular Prolotherapy and Intra-Articular Prolotherapy Alone in Patients With Knee Osteoarthritis and Effusion: A Randomized Clinical Trial, Scientific Journal of Rehabilitation Medicine. (2022) 11, no. 4, 590–600.

[27] Ebadi S. , Karimzad Y. , Aflakian N. , Forogh B. , Mansoori K. , and Babaei-Ghazani A. , Extracorporeal Shockwaves Therapy Versus Corticosteroid Injection for the Treatment of Non-Calcific Rotator Cuff Tendinopathies: A Randomized Trial, Current Orthopaedic Practice. (2023) 34, no. 4, 185–190, 10.1097/bco.0000000000001206.

[28] Ahadi T. , Yousefi A. , Sajadi S. , Yousefi N. , and Babaei-Ghazani A. , Comparing Radial Extracorporeal Shockwave Therapy and Corticosteroid Injection in the Treatment of Piriformis Syndrome: A Randomized Clinical Trial, Journal of Bodywork and Movement Therapies. (2023) 33, 182–188, 10.1016/j.jbmt.2022.09.020. 36775517

[29] Arias-Vázquez P. I. , Guzzardo M. N. , Guzzardo D. R. , Castillo-Avila R. G. , Tovilla-Zárate C. A. , and Ake Montiel M. A. N. , Efficacy of Ozone Injections for Reducing Musculoskeletal Pain in Comparison with Corticosteroid Injections: A Systematic Review and Meta-Analysis, Journal of Back and Musculoskeletal Rehabilitation. (2024) 37, no. 4, 821–838, 10.3233/bmr-230173. 38277280

[30] Hao C. , Yin J. , Liu H. , Cheng Z. , Zeng Y. , and Jin Y. , Pain Reduction and Changes in Serum Cortisol, Adrenocorticotropic Hormone, and Glucose Levels After Epidural Injections With Different Doses of Corticosteroid, Pain Physician. (2024) 27. 38285044

[31] Park D. and Chang M. , The Mechanism of Action of Pulsed Radiofrequency in Reducing Pain: A Narrative Review, Journal of Yeungnam Medical Science. (2022) 39, no. 3, 200–205, 10.12701/jyms.2022.00101. PMC9273139 35385898

[32] Ulusoy G. , Bilge A. , and Öztürk Ö. , Comparison of Corticosteroid Injection and Ozone Injection for Relief of Pain in Chronic Lateral Epicondylitis, Acta Orthopaedica Belgica. (2019) 85, no. 3, 317–324. 31677627

[33] Mohamadi A. , Chan J. , Claessen F. , Ring D. , and Chen N. , Corticosteroid Injections Give Small and Transient Pain Relief in Rotator Cuff Tendinosis: A Meta-Analysis, Clinical Orthopaedics and Related Research. (2017) 475, no. 1, 232–243, 10.1007/s11999-016-5002-1. PMC5174041 27469590

[34] Babaei-Ghazani A. , Fadavi H. R. , Eftekharsadat B. et al., A Randomized Control Trial of Comparing Ultrasound-Guided Ozone (O2-O3) Vs Corticosteroid Injection in Patients With Shoulder Impingement, American Journal of Physical Medicine and Rehabilitation. (2019) 98, no. 11, 1018–1025, 10.1097/phm.0000000000001240. 31188145

[35] Lee S.-U. , Diagnosis and Non-Operative Treatment of Shoulder Pain, Journal of the Korean Medical Association. (2019) .

[36] Freeman J. W. , Empson Y. M. , Ekwueme E. C. , Paynter D. M. , and Brolinson P. G. , Effect of Prolotherapy on Cellular Proliferation and Collagen Deposition in MC3T3-E1 and Patellar Tendon Fibroblast Populations, Translational Research. (2011) 158, no. 3, 132–139, 10.1016/j.trsl.2011.02.008. 21867978

[37] Babaei-Ghazani A. , Moradnia S. , Azar M. et al., Ultrasound-Guided 5% Dextrose Prolotherapy Versus Corticosteroid Injection in Carpal Tunnel Syndrome: A Randomized, Controlled Clinical Trial, Pain Management. (2022) 12, no. 6, 687–697, 10.2217/pmt-2022-0018. 35848821

[38] Karim S. A. , Hamid M. , Choong A. , Ooi M. , and Usman J. , Effects of Platelet-Rich Plasma and Prolotherapy on Supraspinatus Tendinopathy: A Double Blind Randomized Clinical Trial, The Journal of Sports Medicine and Physical Fitness. (2023) 63, no. 5, 674–684. 10.23736/S0022-4707.22.14376-8 37132278

[39] Trebinjac S. and Kitchbi H. , Long-Term Effect of Prolotherapy on Symptomatic Rotator Cuff Tendinopathy, Journal of Health Science. (2015) 5, no. 3, 93–98, 10.17532/jhsci.2016.257.

[40] Mofrad M. K. , Rezasoltani Z. , Dadarkhah A. , Mofrad R. K. , Abdorrazaghi F. , and Azizi S. , Periarticular Neurofascial Dextrose Prolotherapy Versus Physiotherapy for the Treatment of Chronic Rotator Cuff Tendinopathy: Randomized Clinical Trial. JCR, Journal of Clinical Rheumatology. (2019) . 10.1097/RHU.0000000000001218 32975923

[41] Chi H. , Wen X. , Liu X. et al., The Effects of Ozone Therapy on the Expression of IL-8, IL-10, CXCL9 and CXCL11 in Joint Fluid in Patients With Knee Osteoarthritis, Journal of Pharmaceutical Research International. (2022) .

[42] Bocci V. , Borrelli E. , Zanardi I. , and Travagli V. , The Usefulness of Ozone Treatment in Spinal Pain, 2015. 10.2147/DDDT.S74518 PMC4440430 26028964

[43] Burger M. , Africa C. , Droomer K. et al., Effect of Corticosteroid Injections Versus Physiotherapy on Pain, Shoulder Range of Motion and Shoulder Function in Patients With Subacromial Impingement Syndrome: A Systematic Review and Meta-Analysis, South African Journal of Physiotherapy. (2016) 72, no. 1, 1–9, 10.4102/sajp.v72i1.318. PMC6093128 30135893

[44] Sabaah H. M. A. E. and Nassif M. A. J. E. R. , Rehabilitation. What is Better for Rotator Cuff Tendinopathy: Dextrose Prolotherapy, Platelet-Rich Plasma, or Corticosteroid Injections? A Randomized Controlled Study, Egyptian Rheumatology and Rehabilitation. (2020) 47, 1–7.

[45] Foula A. S. , Sabry L. S. , Elmulla A. F. , Kamel M. A. , and Hozien A. I. , Ultrasound-Guided Shoulder Intraarticular Ozone Injection Versus Pulsed Radiofrequency Application for Shoulder Adhesive Capsulitis: A Randomized Controlled Trial, Pain Physician. (2023) 26, no. 4, E329–E340. 37535775

[46] Catapano M. , Zhang K. , Mittal N. , Sangha H. , Onishi K. , and de Sa D. , Effectiveness of Dextrose Prolotherapy for Rotator Cuff Tendinopathy: A Systematic Review, Pm&r. (2020) 12, no. 3, 288–300, 10.1002/pmrj.12268. 31642203

[47] Atar M. Ö. , Korkmaz N. , Aslan S. et al., Comparison of Ultrasound-Guided Subacromial Corticosteroid and Ozone (O2-O3) Injections in the Treatment of Chronic Rotator Cuff Tendinopathy: A Randomized Clinical Trial, The Korean Journal of Pain. (2022) 36, no. 1, 128–136, 10.3344/kjp.22221. PMC9812695 36533317

[48] Bayram D. and Aşık İ. , The Efficacy of Triple Shoulder Injection With Steroids and Ozone in the Treatment of Chronic Shoulder Pain and Range of Motion Limitation, European Journal of Therapeutics. (2024) 30, no. 5, 652–661, 10.58600/eurjther2227.

[49] Seven M. M. , Koca K. , Akpancar S. et al., Promising Results of Prolotherapy in an Elderly Male With Bilateral Partial Rotator Cuff Lesions, Mental Health. (2016) 48, no. 56.

[50] Fawcett R. , Grainger A. , Robinson P. , Jafari M. , and Rowbotham E. , Ultrasound-Guided Subacromial–Subdeltoid Bursa Corticosteroid Injections: A Study of Short-and Long-Term Outcomes, Clinical Radiology. (2018) 73, no. 8, 760-e7–760.e12, 10.1016/j.crad.2018.03.016. 29759589

